# Rhododendron and Japanese Knotweed: invasive species as innovative crops for second generation biofuels for the ionoSolv process†

**DOI:** 10.1039/d1ra01943k

**Published:** 2021-05-21

**Authors:** Louis M. Hennequin, Karen Polizzi, Paul S. Fennell, Jason P. Hallett

**Affiliations:** Department of Chemical Engineering, Imperial College London Exhibition Road London UK SW7 2AZ P.fennell@imperial.ac.uk j.hallett@imperial.ac.uk

## Abstract

We investigated the potential of two terrestrial biomass invasive species in the United-Kingdom as lignocellulosic biofuel feedstocks: Japanese Knotweed (*Fallopia japonica*) and Rhododendron (*Rhododendron ponticum*). We demonstrate that a pretreatment technique using a low-cost protic ionic liquid, the ionoSolv process, can be used for such types of plant species considered as waste, to allow their integration into a biorefinery. *N*,*N*,*N*-Dimethylbutylammonium hydrogen sulfate ([DMBA][HSO_4_]) was able to fractionate the biomass into a cellulose-rich pulp and a lignin stream at high temperatures (150–170 °C) and short reaction times (15–60 minutes). More than 70–80% of the subsequent cellulose was hydrolysed into fermentable sugars, which were fermented into the renewable energy vector bioethanol.

## Abstract 2

Japanese Knotweed (*Fallopia japonica*) and Rhododendron (*Rhododendron ponticum*), two invasive species in the UK that are an environmental threat and economic burden, can be integrated into a flexible ionic liquid based biorefinery process to produce bioenergy and chemicals.

## Graphical abstract

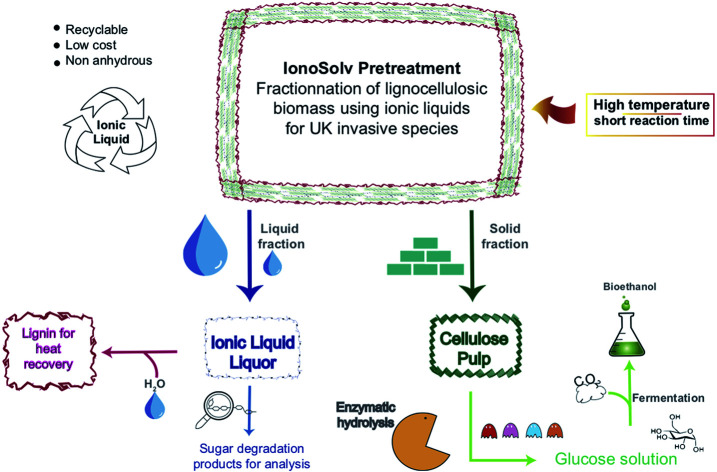

## Introduction

Lignocellulosic or ‘woody’ biomass is the most abundant source of renewable feedstock on Earth. It can be processed and transformed into energy, fuels and chemicals. Unlike their starchy and sugary analogues (sugar cane, beets, also called first generation biofuels), woody feedstocks do not compete with food crops for land use, or require less fertiliser inputs, so their environmental footprint can be reduced.^1^ Nevertheless, both first and second generation biofuels also come with challenges related to sustainability of bioenergy production and use and their role in climate change mitigation. In particular, the impact of bioenergy production on land use and availability was highlighted by the special report of the International Panel on Climate Change in 2019.^2^ Increase in bioenergy use can have significant effects on food security (competition for land), biodiversity and ecosystems (intensive deforestation) and resources (water and nutrients in soils).^3,4^ One way to ensure such bioenergy production in a sustainable manner is to use marginal areas or/and innovative biomass resources such as lignocellulosic waste and residues.^5^

Invasive Non-Native Species (INNS) were recognised as one of the 5 top threats to the natural environment in the United Kingdom.^6,7^ They not only have damaging consequences on natural habitats but also have socioeconomic repercussions on human activities, to the extent that residues from such biomass are a controlled waste in the UK.^8^ Terrestrial plant invasive species are diverse, but they do exhibit one common chemical characteristic: they are comprised of lignocellulosic biomass.^9^ Amongst them, Japanese Knotweed (JPK) and Rhododendron (RHDN), are two of the most common INNS in the United Kingdom under the Wildlife and Countryside Act of 1981 (schedule 9).^10^ Policies involving woody invasive species have been discussed in North America, with more attention to bio-invasion of current species, caution around risk management, and transport.^11,12^ In addition to legal and logistical concerns, another barrier to using invasive species for bioenergy is the biorefinery limitations, which are essentially made for one specific type of feedstock, and the technologies would not be flexible enough to integrate a variety of woody species.^13,14^

All lignocellulosic feedstocks exhibit a more complex molecular structure than their first generation analogues: they are made of a matrix of three polymers, in varying proportions: cellulose, hemicellulose and lignin. The presence of lignin in the plant hinders the subsequent transformation of the polysaccharides into a fuel, *e.g.* bioethanol. Specifically, lignin prevents enzymes from hydrolysing the polysaccharides into their monomers, which would be fed to microorganisms for final fermentation into bioethanol. A pretreatment step is needed for lignocellulosic feedstocks to first separate the different components of the matrix.

Numerous pretreatment methods have been developed for such purposes: physical (ball milling, extrusion), chemical treatments (Organosolv, alkaline) or biological techniques.^15–18,31^ Some involve the use of ionic liquids, which are designer solvents made of an anion and a cation and can be synthesised to be very task specific.^19^ Ionic liquid based pretreatments have been used successfully on a variety of dedicated energy crops or agricultural waste to produce bioenergy.^19–21^ There are several approaches for utilising lignocellulosic biomass and a wide range of ionic liquids can be used.^21–25^ The dissolution process uses ionic liquids, with a strong hydrogen bond basicity anion (often imidazolium based), for the whole dissolution of the biomass and subsequent isolation of the compounds.^23,26–30^ The ‘one pot process’ uses biocompatible ionic liquids such as ethanolamine acetate.^32–35^ The biomass is pretreated and the slurry can be saccharified with no pH adjustment.^36^ Finally, the fractionation process selectively dissolves lignin and hemicellulose, leaving a cellulose pulp, which can be washed and further utilised; and a lignin stream.^37–43^ The ionoSolv pretreatment is a fractionation process, which uses acidic low-cost protic ionic liquids to deconstruct lignocellulosic biomass by selectively dissolving the lignin and the hemicelluloses, leaving a rich cellulose pulp, and recovering lignin with an antisolvent.^37^ It has been used successfully on a wide range of biomass feedstocks: grasses, softwoods and hardwoods.^44–47^

Efforts to investigate and study invasive species as a biofuel feedstock are still scarcely present in the scientific literature, mainly for African and North American species.^48–50^ Here, we demonstrate that the ionoSolv process is feedstock agnostic, and provide characterisation of two UK invasive species as lignocellulosic feedstocks. These species can be integrated into a flexible biorefinery process to produce revenue from species that are an environmental threat and economic burden, contributing to the incentive to remove them from a site.

## Experimental

### Feedstock

Japanese Knotweed dried stems were supplied by the Japanese Knotweed Ltd. Rhododendrons were collected by Mrs T. Dancey from a public area at a roadside in Dorset. All material was air-dried, ground and sieved (180–850 μm).

### Ionic liquid synthesis

*N*,*N*,*N*-Dimethylbutylamine was purchased from Sigma Aldrich with a purity of >99%, 5 mM sulfuric acid. All commercial reagents and solvents were used as received unless otherwise stated.

*N*,*N*,*N*-Dimethylbutylammonium hydrogen sulfate, [DMBA][HSO_4_] was synthesised in a one-step acid-based reaction in equimolar conditions. One mole of the amine, *N*,*N*,*N*-dimethylbutylamine (101.19 g), was weighed into a 500 mL round-bottom flask and cooled with an ice bath. Under stirring, an equimolar amount of the required acid (200 mL of 5 M H_2_SO_4_), was added dropwise. Excess water was removed using a rotary evaporator (Büchi). The water content of the ionic liquid was determined using a volumetric Karl Fisher titrator (V20 Mettler-Toledo).

^1^H NMR: *δ*_H_ (400 MHz, DMSO-d_6_)/ppm: 9.24 (s, 1H, N–H), 3.02 (dt, *J* = 12.9, 5.0 Hz, 2H, N–CH_2_), 2.76 (d, *J* = 4.3 Hz, 6H, N–(CH_3_)_2_), 1.64–1.51 (m, 2H, N–CH_2_–CH_2_), 1.30 (h, *J* = 7.4 Hz, 2H, N–CH_2_–CH_2_–CH_2_), 0.89 (t, *J* = 7.4 Hz, 3H, N–CH_2_–CH_2_–CH_2_–CH_3_). ^13^C NMR *δ*_C_ (101 MHz, DMSO-d_6_)/ppm: 56.62 (N–CH_2_), 42.48 (N–CH_3_), 25.82 (N–CH_2_–CH_2_), 19.40 (N–CH_2_–CH_2_–CH_2_), 13.71 (N–CH_2_–CH_2_–CH_2_–CH_3_).

### Compositional analysis

Compositional analysis was carried out according to a published procedure by the National Renewable Energy Laboratory (NREL).^51^ Briefly, 300 mg (calculated on dry matter basis) of biomass or recovered pulp was incubated with 3 mL of 72% sulfuric acid at 30 °C for 1 hour and then diluted with 84 mL distilled water before being autoclaved for 1 hour at 121 °C. The samples were then filtered through filtering ceramic crucibles of a known weight with filtrate used to determine acid soluble lignin by UV analysis at 240 nm and carbohydrate concentrations. The determination of the total sugar content was carried out using an HPLC system with a Refractive Index Detector (RID) (Shimadzu, Aminex HPX-87P from Bio rad, 300 × 7.8 mm, purified water as mobile phase at 0.6 mL min^−1^, column temperature 85 °C, 25 minute run).

The crucibles were oven dried at 105 °C overnight before weighing to determine acid insoluble lignin and then incubated at 575 °C in a muffle furnace to determine ash content, as described in the NREL protocol. The reported lignin content is the sum of the acid soluble and acid insoluble lignin contents.

The ionic liquid liquor compositional analysis was carried out using a Shimadzu HPLC system (RID, Aminex HPX-87H Bio rad column, 300 × 7.8 mm) with 0.005 M H_2_SO_4_ as mobile phase (0.6 mL min^−1^). The column temperature was 55 °C and the time acquisition was 35 min.

### Fractionation of biomass

The pretreatment of the biomass was conducted as described by the ionoSolv standard operating procedure from the Hallett Laboratory.^52^ All samples were run in triplicates. The lignocellulosic biomass was pretreated with the ionic liquid with a final water content of 20 wt%, taking into account the water content of the IL and the moisture content of the biomass. The solvent to biomass ratio used was 1 : 5 g g^−1^ on an oven dried basis. The biomass was then pretreated for a set time and temperature in a convection oven. The pretreated pulp fraction was then washed by Soxhlet overnight to remove any residual ionic liquid using ethanol (VWR). The pulp was weighed and pulp yield was calculated on an oven dried basis. The lignin dissolved within the ionic liquid liquor fraction was precipitated by the addition of an anti-solvent: 1 g of water for 3 g of biomass used. After centrifugation, the ionic liquid liquor was recovered and separated from the lignin. The lignin was dried using a freeze dryer (LabConco FreezeDry Benchtop) and the lignin yield was calculated on an oven dried basis.

On the basis of compositional analysis of both untreated samples and pulps from the ionoSolv process, glucan recovery, hemicelluloses removal and delignification were determined according to eqn (1)–(3) respectively:

1 Glucan recovery = (glucan_pulp_ × yield_pulp_)/glucan_untreated_

where glucan_pulp_ is the glucan content in the cellulose pulp, yield_pulp_ is the yield of the pulp after ionoSolv processing relative to untreated biomass, and glucan_untreated_ is the glucan content in the untreated biomass.

2 Hemi removal = (hemi_untreated_ − hemi_pulp_ × yield_pulp_)/hemi_untreated_

where hemi_untreated_ is the hemicellulose content of untreated biomass calculated as the sum of all sugars (except glucose) and hemi_pulp_ is the hemicelluloses content in the cellulose pulp.

3 Delignification = (lignin_untreated_ − (lignin_pulp_ × yield_pulp_))/lignin_untreated_

where lignin_untreated_ is the lignin content in the untreated biomass and lignin_pulp_ is the lignin content in the recovered cellulose pulp after the ionoSolv pretreatment.

### Saccharification assay

Enzymatic saccharification was carried out according to an adapted procedure developed by the NREL in triplicates.^53^ The saccharification was carried out on air dried samples at 50 °C and 250 rpm for 7 days, according to the NREL protocol and previous research done in the Hallett Laboratory.^54,55^ Briefly, 100 ± 10 mg of pretreated and un-pretreated samples were incubated in a medium consisting of 5 mL of 1 M sodium citrate buffer at pH 4.8, 40 μL of tetracycline antibiotic solution, 30 μL cycloheximide antibiotic solution, 40 μL of CTec2 enzyme mix from Novozymes and purified water to obtain a final volume of 10 mL. Blank samples used 100 μL of water instead of biomass to correct for any sugar background concentration. Sugar concentrations were obtained after filtering 1 mL of the hydrolysate through a 0.2 μm PTFE syringe filter. Samples were run on a Shimadzu HPLC system (RID, Aminex HPX-87P Bio rad column, 300 × 7.8 mm) with purified water as the mobile phase (0.6 mL min^−1^) at 85 °C for 20 minutes. The conversion of glucose was then calculated according to the NREL protocol, taking into account the anhydrous correction factors of the polysaccharides, the pulp yields from the pretreatment and the glucan content of the untreated biomass from compositional analysis.

### Elemental analysis

The CHNS analysis was performed using a Vario Micro tube supplied by Elementar, consisting of a dynamic flash combustion analysis and thermal conductivity detection. Accuracy is ±0.30% absolute. Oxygen content was obtained by difference.

The Higher Heating Value (HHV) in MJ kg^−1^ was calculated according to the Demirbas formula (an adaptation of Dulong's formula)^56^ on a dry ash free basis, according to eqn (4):

4 HHV = 33.5 × C + 142.3 × H − 15.4 × O − 14.5 × N

where C = carbon content, H = hydrogen content, O = oxygen content, and N = nitrogen content.

### Gel permeation chromatography (GPC)

GPC measurements were performed using an Agilent 1260 Infinity instrument equipped with a Viscotek column set (AGuard, A6000M and A3000M). The Agilent 1260 Infinity RID detector was used for detection. HPLC grade DMSO containing 1 g L^−1^ of Lithium Bromide was used was the effluent at 0.4 mL min^−1^ at 60 °C. Samples were prepared by dissolving 20 mg lignin in 1 mL eluent and filtered through a 0.2 μm syringe filter. Ten pullulan standards (Agilent calibration kit, 180 < Mp < 780 000) were used for calibration. GPC was used on the lignin to determine their molecular distributions and absolute molar masses (*M*_w_ and *M*_n_ and polydispersity *Đ*, where *Đ* = *M*_w_/*M*_n_).

### Fermentation

Solutions with sufficient concentration of glucose (around 20 mg mL^−1^) were obtained by carrying out enzymatic saccharification over 24 hours with high enzyme loading. 330 mg of pulp samples or 1.6 g untreated samples (dry matter) were incubated with 4 mL of sodium citrate buffer at pH 4.8, 4 mL of purified water and 400 μL of CTec2 enzyme mix from Novozymes. Saccharified samples were then centrifuged to remove the solids and the resulting glucose containing solutions were used to make a medium by mixing with 1× yeast nitrogen base (YNB) with amino acids. The concentration of the resulting solutions was checked using the HPLC system (Shimadzu, RID detector, Aminex HPX-87P from Bio rad).

Single colonies (OD_600_ of 0.1) of *Saccharomyces cerevisiae* BY4741 [MATa his3Δ1 leu2Δ0 met15Δ0 ura3Δ0] were used for the fermentation studies on the hydrolysate solutions. The fermentation was carried out in triplicates at 30 °C with shaking at 250 rpm for 48 hours in an incubator (Labnet International). After the fermentation, the optical density of the cultures was measured to check for any abnormal high density due to bacterial development. The supernatant was analysed using an HPLC system (RID detector, Aminex HPX-87H Bio rad column, 300 × 7.8 mm, 0.005 M H_2_SO_4_ as mobile phase, 0.6 mL min^−1^ for 35 minutes at 55 °C) for ethanol concentration. Yields of ethanol were standardized to the amount of biomass used for each sample to obtain the hydrolysates.

## Results and discussion

### Compositional analysis of untreated biomass

**Fig. 1:**
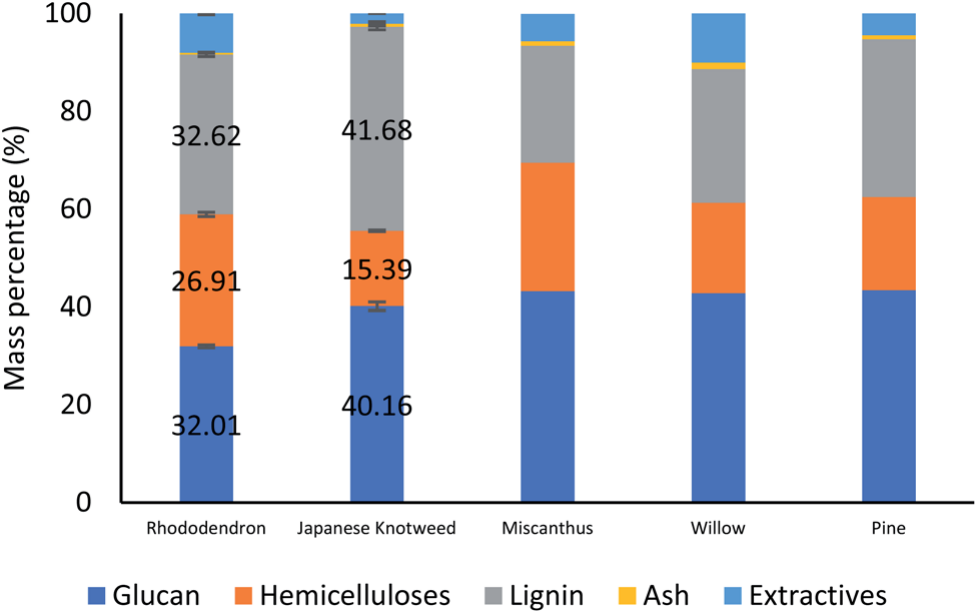
Compositional analysis of the Japanese Knotweed and Rhododendrons used for this study, shown alongside other typical biofuel feedstocks.^45–47^

Japanese Knotweed (JPK) and Rhododendron (RHDN) samples were first analysed for their glucan, hemicellulose and lignin content (Fig. 1), which have not been previously reported in the literature. The results show that JPK has the highest glucan and lignin contents between the two feedstocks with 40.2% and 32% respectively, with a lower hemicellulose content of 15.4%. Rhododendron exhibits a higher hemicellulose content than JPK at 26.9%, with lower glucan and lignin. These values can be compared to typical biofuel crops reported in the literature and used for the ionoSolv process as seen in Fig. 1: a grass such as *Miscanthus* with a glucan content of 43.2%, a softwood such as pine (*S. sylvestris*) 43.4% or a hardwood such as willow (*Salix* spp).^45,46^ The results of the compositional analysis of JPK are close to that for pine, with a high glucan (above 40%) and lignin content (30–40%) and lower hemicelluloses. On the other hand, RHDN exhibits similar composition to a hardwood such as willow with more distributed portions of the polymers. These results show that these invasive species exhibit a composition comparable to those suitable for second generation of biofuels and reported in the literature.^46^

Given the high lignin content of the feedstocks at 32.6% and 41.7% for RHDN and JPK respectively, if the cellulose is to be prepared for subsequent enzymatic hydrolysis and fermentation into a biofuel, they will require a pretreatment step.

### Biomass deconstruction and cellulose digestibility

The ionic liquid *N*,*N*,*N*-dimethylbutylammonium hydrogen sulfate – [DMBA][HSO_4_] – was selected for its easy synthesis consisting of an equimolar acid–base reaction between two inexpensive starting chemicals; but also for its performance for quantitative sugar release from pine compared to other protic ionic liquids.^46^ The price range of [HSO_4_]^−^ based ionic liquids was estimated between $0.78 to $5.88 kg^−1^, which is competitive with organic solvents such as acetone or ethyl acetate.^57,58^

**Fig. 2:**
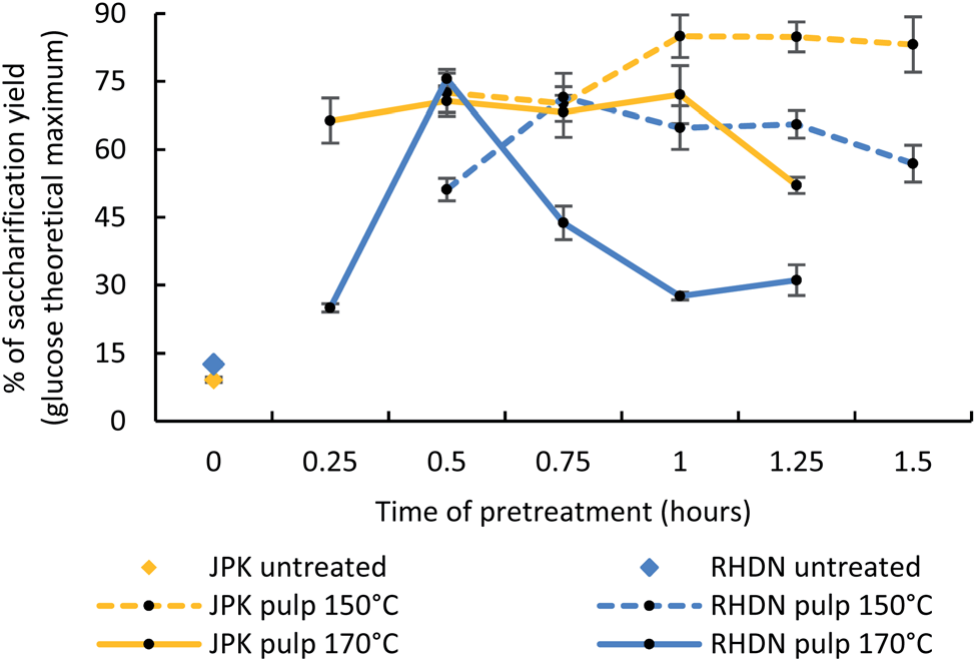
Enzymatic hydrolysis conversion of untreated samples and the pulp obtained from the pretreated pulps at 150 °C and 170 °C.

The parameters of the ionic liquid pretreatment can be tuned depending on the biomass and the preferred outcome of the pretreatment. In this study, we investigated the effect of time and temperature of the treatment with time course pretreatments (varying of time and temperature of the biomass–ionic liquid reaction) and analyse how these species respond to ionoSolv fractionation. The glucose release after pretreatment from the pulp was selected as an assessment of the success of the pretreatment, in order to produce fermentable glucose solutions for conversion into bioethanol, chosen an end-use example of the cellulose rich pulp in this study. This is a key value in determining the accessibility of the glucose by enzymes and therefore the deconstruction of the lignocellulosic matrix from the pretreatment (as lignin hinders glucose accessibility). As shown in Fig. 2, the enzymatic hydrolysis of non-treated samples for the two invasive species only yielded 12.6% and 9.2% of glucose for RHDN and JPK respectively. This shows that the presence of lignin does hinder the access of enzymes to the cellulose and the need for pretreatment prior to subsequent utilisation of the cellulose.

Time courses at 150 °C and 170 °C were performed, based on the compositional analysis of the feedstocks studied here, and previous research carried out for woody samples, involving temperatures above 120 °C combined with shorter reaction times (less than 2 hours).^45–47,59^ The trends concerning the pulp and the lignin yields are available in the ESI (Fig. S1†).

The maximum glucose release for RHDN was achieved at 30 minutes and 170 °C with 75.6% glucose (a 6-fold increase compared to the untreated tests) and 71.5% for 45 minutes at 170 °C. This is similar to what has been reported with pretreatment of pine using [DMBA][HSO_4_] with sugar releases over 70% at high temperature and short reaction time (170 °C, 30 min).^46^ After reaching the optimal value, the glucose yields decrease at both temperatures. This indicates either degradation of the cellulose, charring of the pulp at harsher pretreatment conditions, or re-deposition of pseudo-lignin (hydrolysed smaller fragments of lignin, possibly re-condensed with degradation products) on to the surface of the pulp during the process.^47^ Shorter reaction times are preferable for a pretreatment process (since this reduces equipment size and cost); this is why the optimal pretreatment condition was subsequently considered to be 170 °C for 30 minutes for RHDN for further analyses.

The glucose release from JPK at 170 °C after pretreatment was consistently lower than at 150 °C. This indicates that 170 °C is too harsh for pretreating JPK. This is consistent with the composition of the biomass and differences between the two feedstocks. JPK has a greater glucan content than RHDN; higher temperatures would degrade the glucose faster, while solubilising the lignin is achieved at lower temperatures. The highest glucose release for JPK was observed at 150 °C for 1 hour at 85%, a 7.7-fold increase compared to untreated samples.

The pulps at 150 °C for 1 hour and 170 °C for 30 minutes were selected for further analysis for JPK and RHDN respectively.

### Cellulose pulp composition

After the pretreatment, three fractions are obtained: a cellulose-rich pulp, lignin precipitated from the liquor and an ionic liquid liquor.

**Fig. 3:**
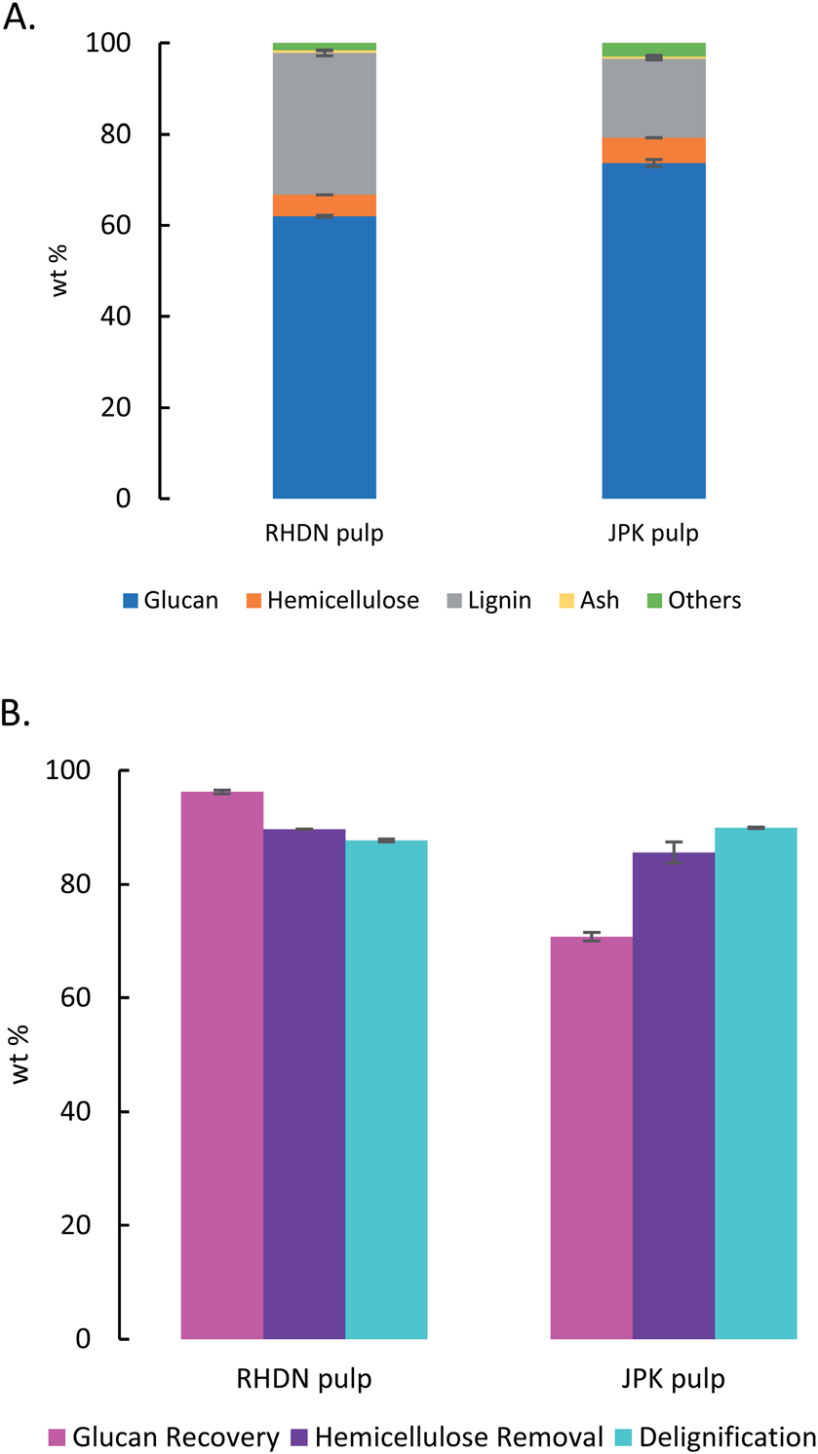
(A) Compositional analysis of the pulps recovered from the pretreatment (170 °C 30 min for RHDN, 150 °C 1 h for JPK). (B) Analysis of the pulp from the pretreatment (170 °C 30 min for RHDN, 150 °C 1 h for JPK). Values are in weight percentage.

The compositions of the pulps were determined to assess the cellulose purity and effect of the ionic liquid on the lignocellulosic matrix. The results are shown in Fig. 3. Both pulps still retain some lignin, since the delignifications are just below 90%. This level of lignin extraction in the pulps is enough to deconstruct the biomass and achieve significant saccharification yields as shown in Fig. 2 (85% for JPK, 75.6% for RHDN). The lignin content of the RHDN pulp is higher than in the untreated samples. This could be due to the deposition of a condensed lignin oligomers (pseudo-lignin), which are water soluble, on to the cellulose-rich pulp at harsh pretreatment conditions (170 °C for RHDN compared to 150 °C for JPK).^60,61^

The pretreatment yields high cellulose purity of the pulps: 62% for RHDN and over 73% for the JPK, which represents a glucose recovery of 96.2% and 70.8% respectively. Even though the glucose recovery is lower for the JPK, which has a higher initial glucan content (Fig. 1), the cellulose purity of the pulp is higher. This could be due to hydrolysis of the less crystalline regions of cellulose during pretreatment.^62^ Overall, the ionic liquid fractionated the biomass during the pretreatment, leaving a rich cellulose pulp with around 5% of hemicelluloses and some lignin, which has been disrupted.

### Lignin analysis

Following the pretreatment, the lignin and the hemicelluloses are solubilised in the ionic liquid liquor. Lignin being insoluble in water, it can be precipitated from the liquor by the addition of water as an anti-solvent. Extensive study of the ionoSolv lignin structure was not in the scope of this study, and has been done elsewhere.^25,63^ Here, elemental analysis was performed to assess the higher heating value of the ionoSolv lignin for incineration, while gel permeation chromatography was used to determine the molecular size and weight of the lignin extracted from the pretreatment.

The Higher Heating Values (HVV) of the lignins from the best performing pulps were calculated and shown in Table 1 (the values for the lignin for all the time courses are shown in Fig. S2†). The HHV for the untreated biomass is 16.3 MJ kg^−1^ for both feedstocks. This is within the range of the values reported in the literature for other biomass feedstocks (8–20 MJ kg^−1^) or bio oils (16–19 MJ kg^−1^).^64,65^ The lignin extracted from the ionoSolv process exhibits a 1.5-fold increase compared to untreated raw biomass. This is due to the higher carbon and lower oxygen contents of the lignin compared to untreated biomass.^66,67^ ionoSolv lignin can be burned for energy as a fuel, with more energy liberated than untreated biomass, contributing to the valorisation of all products derived from the pretreatment of the biomass.

**Table 1:** Higher Heating Values (MJ kg^−1^), average molecular weight *M*_n_ (Da), number molecular weight *M*_w_ (Da), and polydispersity (*Đ*) of the samples studied

Sample	HHV	*M*_n_	*M*_w_	*Đ*
RHDN ionoSolv lignin (170 °C 30 min)	24.50	1291	6321	5.1
JPK ionoSolv lignin (150 °C 1 h)	23.28	1064	5040	4.67
RHDN untreated biomass	16.34			
JPK untreated biomass	16.31			

If not burned for energy, lignin can also be used as a precursor for value added chemicals, for which characterisation of the lignin is often needed.^17,42^ The GPC results determined the average molecular weight (*M*_n_), the number molecular weight (*M*_w_) and the dispersity (*Đ*) was calculated for the lignin extracted from the pretreatment (Table 1). We can observe a trend of the *Đ* is higher for RHDN than JPK: as the time of pretreatment increases, so does the *Đ*. This is due to the higher cleavage of lignin ether bonds in harsher pretreatment conditions.^68^ This trend has been observed for other lignocellulosic feedstocks such as Miscanthus and Willow with protic ionic liquids and similar pretreatment conditions.^45,47^ Higher temperatures tend to accelerate lignin condensation reactions with fragments of lignin and possibly degradation products (pseudo-lignin). The values for both *M*_n_ and *M*_w_ for RHDN seems to be higher than the ones reported for pine using the same conditions (*M*_n_ 775 Da, *M*_w_ 4949 Da).^46^

Differences in molecular weight of the lignin are inherent to the species of the biomass itself, but also to the pretreatment technology used, and its severity.^45,69^ The desired properties of ionoSolv lignin from invasive species would depend on its end application. Lignin with higher molecular weight is preferable for composite materials such as fillers, while lower molecular weight lignin is often used in biological routes.^24,70,71^

Different lignin structures and properties between feedstocks and even within feedstocks affect the delignification chemistry in biomass fractionation using ionic liquids.^72^ However, this study demonstrates that the ionoSolv pretreatment can be feedstock agnostic and applied for a variety of different lignocellulosic feedstocks, such as UK invasive species, to extract lignin and generate a cellulose rich pulp.

### Fermentation to bioethanol

**Fig. 4:**
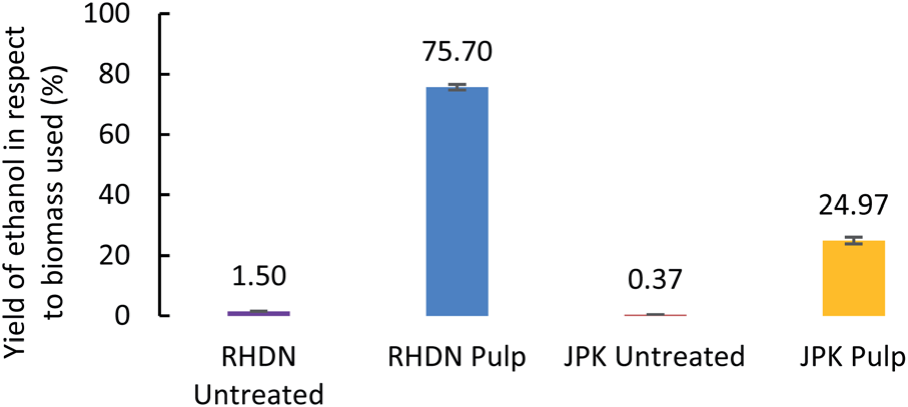
Ethanol yields from the hydrolysate of pretreated pulps (with the highest glucose release), standardised to the amount of biomass used (dry matter).

To demonstrate potential valorisation of the hydrolysate, fermentation of the glucose solutions into bioethanol was performed. The obtained hydrolysates which released the highest glucose yields were used to grow yeast cells (*Saccharomyces cerevisiae*) for ethanol production. Ethanol production was observed for the two feedstocks, as shown in Fig. 4.

The ethanol yields observed for the untreated invasive species are below 2%, much lower than the ones obtained from the pretreated samples, as a result of the high amount of biomass necessary to achieve a sufficient sugar concentration for the fermentation. This further demonstrates the necessity of pretreatment for lignocellulosic biomass and subsequent valorisation into a fuel. The yield of glucose conversion into bioethanol from pretreated RHDN reached 75.7% showing that this feedstock is suitable for fuel production under these conditions. The yield of ethanol from pretreated JPK is slightly below 25%, which is a 67-fold increase compared to untreated JPK. The significant difference between the fermentation of RHDN and JPK can be explained by the presence of fermentation inhibitors in the hydrolysate.^73^ The presence of degradation products from the pretreatment such as furans and carboxylic acids can hinder the fermentation of yeasts.^74^ In fact, the HPLC analysis of solutions after fermentation (Fig. S3 ESI†) show slightly higher concentrations of acetic and levulinic acids for the JPK than for the RHDN; with the presence of formic acid only in the JPK fermentate.

We have shown here that these invasive species samples can be deconstructed using a low-cost innovative solvent and that the cellulose pulp derived from the process can be converted into a biofuel with significant yields, especially for RHDN. Such biomass can be used as a resource to feed into the ionoSolv process and could have a significant positive environmental impact. Instead of being burned as contaminated waste, this biomass could be used to yield renewable and financially attractive compounds using an innovative technology that has proven successful on other feedstocks (softwoods, hardwoods, grasses).

### Technoeconomic considerations

The associated costs of a contractor clearing a site infested with JPK ranges from £7k to £10k, depending on the site. In Great Britain, the estimated total cost of removal and disposal of JPK alone stands at £165 million per year, whereas the current control efforts against RHDN species are estimated to cost over £8 million per year.^10,75,76^ The most common methods advised by DEFRA and the Environmental Agency when dealing with such species include spraying of chemicals, burying or burning the plants. Alternatively, they can be disposed of off-site, usually by hiring a specialist contractor – but these methods are expensive and can have repercussions on the environment.^6–8,77^ The Scottish Government offers rural payment schemes for land users to manage an infested area: £5500 per hectare for manual eradication of an RHDN infestation; land users can claim up to £17 000 for treating with one hectare of a JPK-infested site with herbicide.^77,78^ Through this study, we have shown that a chemical engineering process for biomass can be an alternative to herbicide treatment or waste disposal and that these invasive species can instead be a source of useful products and fuels.

An economic assessment of the ionoSolv process has been reported in the literature using virgin wood at an industrial size.^59^ The results of this study for the invasive species were adapted to assess if an economic profit can be made using this biomass: the pulp and lignin yields for RHDN, the amount of coproducts found in the liquor, *i.e.* furfural and acetic acid, and the amount of RHDN that could be utilised. With RHDN taking up 3.3% of Britain's woodland (98 700 ha), this feedstock represents a potential self-sufficient market for a biorefinery. This could represent a significant increase in land used for bioenergy without competing with food or forests.^79^ JPN would still have value and could be used in co-processing with RHDN. The details of the technoeconomic assessment can be found in the Tables S4 and S5 of the ESI.†

We can assume that most of the capital, energy, water and solvent costs are equivalent for all type of feedstocks at scale for the ionoSolv plant. However, it has been assumed that the feedstocks are “free”, so that the costs of obtaining them are covered by the grants for removing them (and bearing in mind that they would ordinarily need to be disposed of as controlled waste at great expense). Table 2 shows that based on this study, an actual net gross margin could be made from using the aerial parts of such invasive species if they were integrated into an ionoSolv biorefinery.

**Table 2:** Techno-economic considerations of the integration of RHDN as an invasive species into the ionoSolv process at scale (more details can be found in the ESI)

Revenue (per ton of biomass)		Cost (per ton of biomass)	
Pulp	£72	Ionic liquid solvent	£16
Lignin	£33	Water use	£4
Furfural	£40	Capital cost	£13
Acetic acid	£23	Energy	£51
		Biomass	£0
**Total**	**£167**		**£84**
**Net gross margin**	**£83 (50%)**		

We can deduce from the values of Table 2 that the ionoSolv process becomes neutral if a ton of species has a cost at the factory gate of £83 per ton of biomass. This mean would mean that a subsidy that would bring down the price of the feedstock below £83 per ton of invasive species to be sent through the ionoSolv factory, would make a profit. The market price of typical biofuel feedstocks can range between £52 and £60 per ton of biomass (pine, wood pellets, Miscanthus).^80–82^ This shows that such invasive species feedstocks could be used to produce valuable chemicals using the ionoSolv process, with little subsidy from the government; and lead to great savings in government funds currently used to tackle such invasive species from spreading. This work clearly demonstrates that a hazardous material, appropriately managed, can become a revenue stream; and can be integrated into the ionoSolv technology at larger scale.

To the authors' knowledge, this is the first time UK invasive species such as JPK and RHDN were investigated as biofuel feedstocks. This represents an upgrading of an under-utilised biomass, that is very hard to eradicate by chemical and physical means. In combination with strict management practises to avoid further spreading of invasive species (*i.e.* transport, harvest), this could lead to reducing the negative environmental impact of the invasive species by creating renewable energy and therefore a revenue, allowing eradication at a lower cost.

## Conclusion

We determined that the composition of two UK invasive species, Japanese Knotweed and Rhododendron, are comparable to typical biofuel feedstocks, such as pine and willow. The ionoSolv process is capable of processing this biomass at high temperature and short reaction times into value-added bioproducts, demonstrating potential as an energy feedstock. This pretreatment process proves again that it is feedstock agnostic, yielding a very high cellulose rich pulp from these two invasive species, as well as a lignin stream. The cellulose was hydrolysed and successfully fermented into bioethanol, while the lignin was valorised to produce heating energy. Under correct handling and transport, such invasive species could be feed into the ionoSolv process and generate revenue, with a gate fee of £83 per ton. As these feedstocks are easily available and not agriculturally grown on purpose, they have the advantage of take the pressure off land use and land availability for the production of bioenergy.

## Conflicts of interest

There are no conflicts to declare.

## Supplementary Material

RA-011-D1RA01943K-s001
