# Supplementary material for: Rhododendron and Japanese Knotweed: invasive species as innovative crops for second generation biofuels for the ionoSolv process
Source: RSC Adv. 2021 May 21;11(30):18395–403. doi: 10.1039/d1ra01943k (PMC9033429; doi:10.1039/d1ra01943k)
Supplement: RA-011-D1RA01943K-s001 [file RA-011-D1RA01943K-s001.pdf]

Electronic Supplementary Material (ESI) for RSC Advances.
This journal is © The Royal Society of Chemistry 2021

Supplementary information

Rhododendron and Japanese Knotweed: invasive species as innovative crops for second generation biofuels for the ionoSolv process

Louis M. Hennequin[1], Karen Polizzi[1], Paul S. Fennell[1], Jason P. Hallett[1]

[1]Department of Chemical Engineering, Imperial College London, Exhibition Road, London, UK, SW7 2AZ

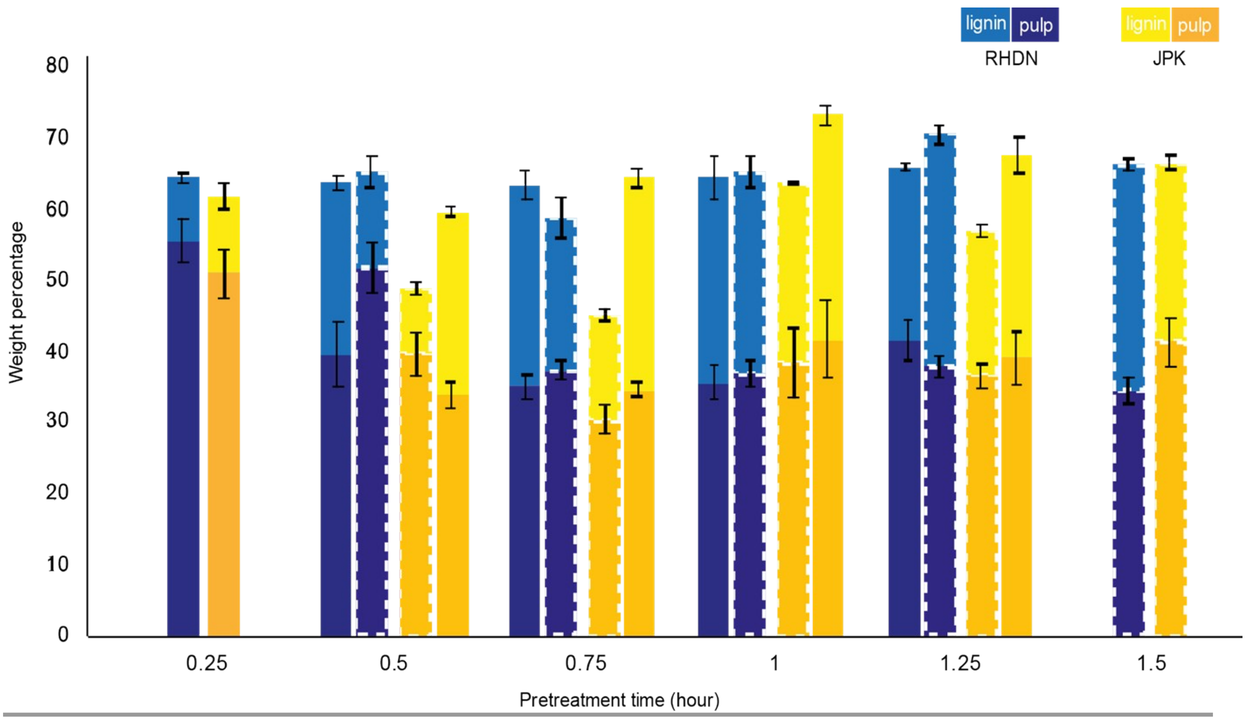

Figure S1 Pulp and lignin yields from the time course pretreatments of JPK and RHDN using DMBA HSO_4 (20%wt water) at 20 wt % biomass:IL loading. Dashed bars indicate pretreatment at 150°C, solid bars at 170°C.

Sample	RHDN - 150°C			RHDN - 170°C		
Time (h)	M_n	M_w	Đ	M_n	M_w	Đ
0.5	1436	6824	4.97	1291	6321	5.10
0.75	1311	7204	5.14	1201	6666	5.56
1	1187	7386	6.02	1166	6226	5.36
1.25	1113	7798	6.83	1131	6287	5.56

A.

Sample	JPK - 150°C			JPK - 170°C		
Time (h)	M_n	M_w	Đ	M_n	M_w	Đ
0.5	1218	4544	3.59	842	4067	4.95
0.75	1050	4063	3.96	824	4322	5.48
1	1064	5040	4.67	990	5484	5.68
1.25	1077	4063	4.12	857	5332	5.96

B.

C.

Figure S2. **A**. GPC results of the lignin. M_n and M_w (Da) for pretreatment of RHDN **B.** GPC results of the lignin. M_n and M_w (Da) for pretreatment of JPK **C**. Higher Heating Values of the lignin obtained after pretreatment using the Demirbas formula ($MJ.kg^{-1}$)

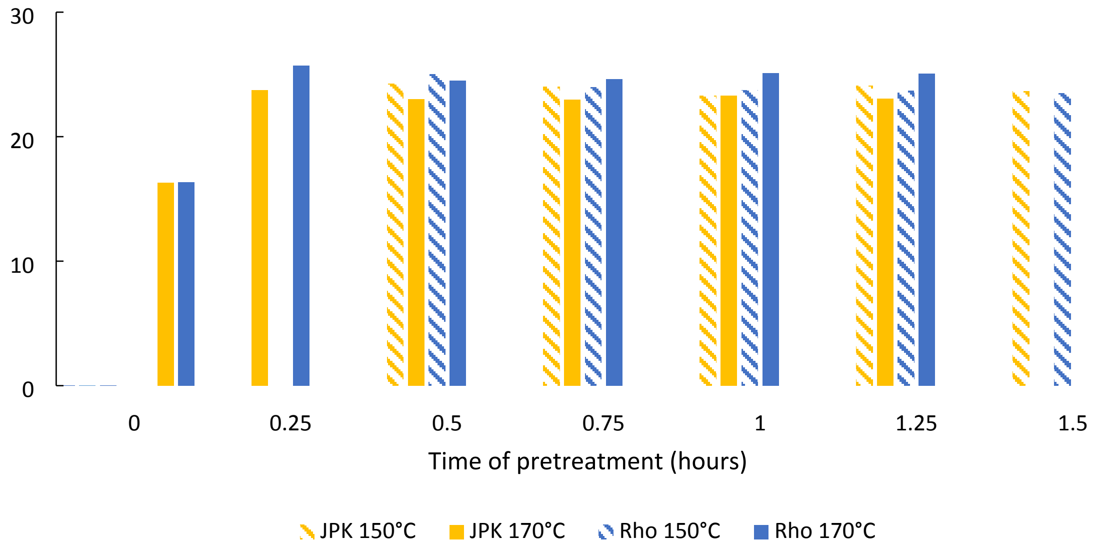

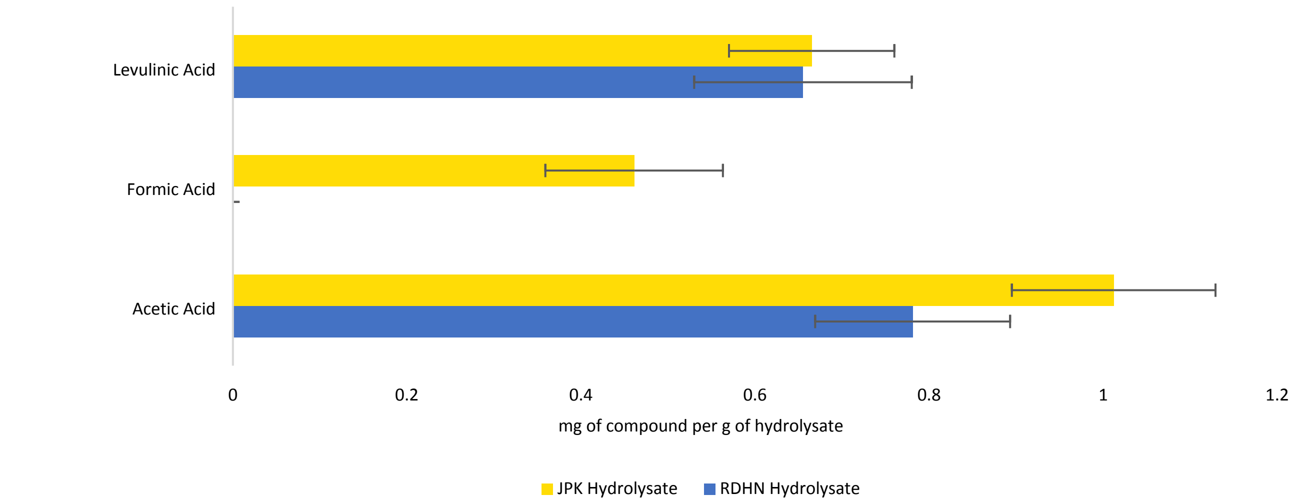

Figure S3 HPLC analysis of the weak acids found in the fermentate for JPK and RHDN

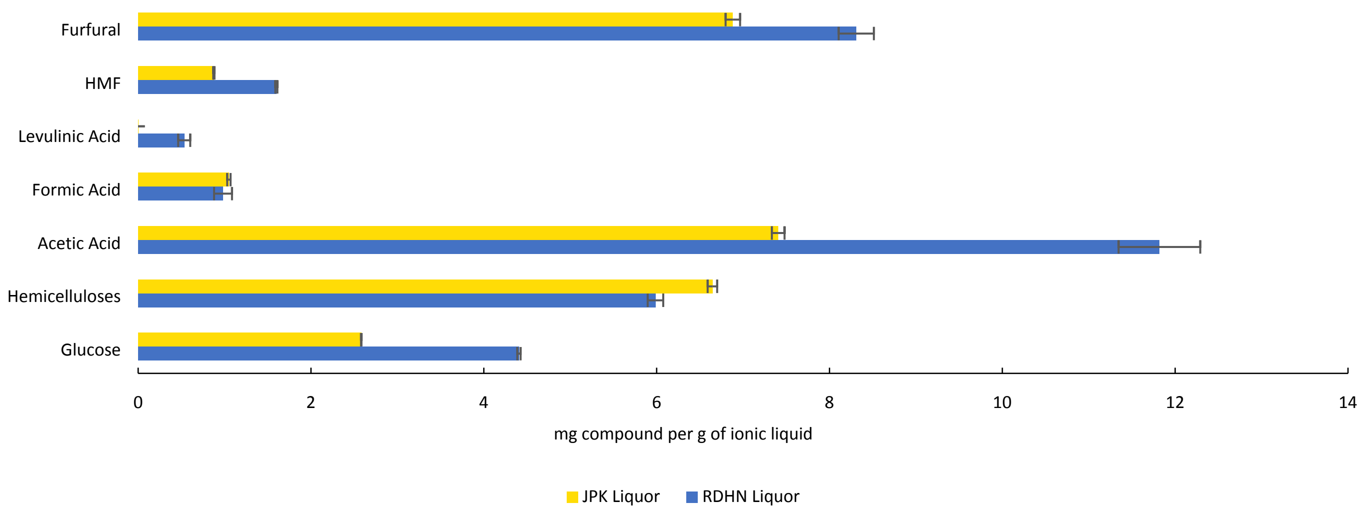

Figure S4 Analysis of the compounds found in the ionic liquid liquor after pretreatment

COST and VALUE
(£ per ton of wood)

	Virgin biomass
Pulp	£72
Lignin	£33
Furfural	£40
Acetic acid	£23
**REVENUE**	**£168**
Solvent	£16
Biomass	£60
Water	£4
Capital	£13
Energy	£51
**COST**	**£145**
**NET**	**£23**
**GROSS MARGIN**	14%

If cost of biomass if £60 / ton

Pulp		£72
Lignin		£33
Furfural		£40
Acetic acid		£23
**REVENUE**		£168
Solvent		£16
Biomass		£60
Water		£4
Capital		£13
Energy		£51
**COST**		£145
**NET**	**£**	**23**
**GROSS MARGIN**		14%

COST INPUTS

**Solvent**		
Price of IL	1.24	$/kg
	0.82	£/kg
Water loading	0.2	wt/wt fraction
Biomass loading	0.2	wt/wt fraction
IL purge rate Virgin	0.005	wt/wt
**Virgin Biomass**		
Gate fee dedicated bionergy crop without any subsidy	60	£/ton
**Water**		
Cost	4	£/ton wood
**Energy**		
Heat	50.8	£/ton wood

VALUE INPUTS

**Pulp**		
Market price	160	£/ton pulp
Yield	0.45	ton pulp/ton wood
Revenue	72	£/ton wood
**Lignin**		
Market price	132	£/ton lignin
Yield	0.25	ton lignin/ton wood
Revenue	33	£/ton wood
**Furfural**		
Market price	1000	£/ton FF
Yield	0.04	ton FF/ton wood
Revenue	40	£/ton wood
**Acetic acid**		
Market price	460	£/tonne
yield	0.05	ton FF/ton wood
Revenue	23	£/ton wood

CAPITAL INPUTS

	Invasive species (RHDN)	Virgin wood	
Plant size	1,000,000	771,000	ton/year
	3030.30	2336.36	ton/day
Operation	330		days/year
Lifetime/depreciation	10		years
Tax rate	0.2	£/£	
Interest rate	0.13	£/£	
Exchange rate	1.5	$/£	

Capital cost estimates

1. Estimate for Innovations

Cost of pilot plant (1 t/day)	250,000		£
	68,388,055	57,005,704	£
Scaled capital cost	9	10	£/ton wood
	26,941	22,457	£/day

2. Estimate with different feedstocks

in £	Wheat straw plant (scaled)	Invasive species plants (scaled)	Virgin wood plant (scaled)	
Size	771,309	1,000,000	771,000	ton
Estimated process cost (no heat integration)	19,278,959	23,121,910	19,273,553	£
Estimated process cost (with heat integration)	67,767,925	81,276,372	67,748,920	£
**Total capital cost (no HI)**	79,043,733	**94,799,832**	**79,021,566**	£
Total capital cost (with HI)	277,848,494	333,233,127	277,770,572	£
**Scaled capital cost (no HI)**	**10**	**12**	**13**	£/ton wood

Table S5 Details of the technoeconomic assessment of the IonoSolv process, adapted for the studied invasive species. Gate fee here fixed at £60 (dedicated bioenergy crop) for comparison.
